# Co-production and conservation physiology: outcomes, challenges and opportunities arising from reflections on diverse co-produced projects

**DOI:** 10.1093/conphys/coaf049

**Published:** 2025-07-18

**Authors:** Steven J Cooke, Nolan N Bett, Scott G Hinch, Chief Bonnie Adolph, Caleb T Hasler, Bradley E Howell, Alexandra N Schoen, Eric J Mullen, Nann A Fangue, Anne E Todgham, Melanie J Cheung, Rachel C Johnson, Rebekah Sze-Tung Olstad, Marine Sisk, Chief Caleen Sisk, Craig E Franklin, Robert C Irwin, Terri R Irwin, Wolfgang Lewandrowski, Emily P Tudor, Hayden Ajduk, Sean Tomlinson, Jason C Stevens, Alana A E Wilcox, Jolene A Giacinti, Jennifer F Provencher, Reyd Dupuis-Smith, Frédéric Dwyer-Samuel, Michelle Saunders, Leith C R Meyer, Peter Buss, Jodie L Rummer, Brittany Bard, Andrea Fuller

**Affiliations:** Department of Biology and Institute of Environmental and Interdisciplinary Science, Carleton University, 1125 Colonel By Dr., Ottawa, ON K1S 5B6, Canada; Department of Forest & Conservation Sciences, University of British Columbia, 2424 Main Mall, Vancouver, BC V6T 1Z4, Canada; Department of Forest & Conservation Sciences, University of British Columbia, 2424 Main Mall, Vancouver, BC V6T 1Z4, Canada; Cayoose Creek Band, 810 Highway 99 North, Lillooet, BC V0K 1V0, Canada; Department of Biology, The University of Winnipeg, 515 Portage Ave., Winnipeg, MB R3B 2E9, Canada; Department of Biology, The University of Winnipeg, 515 Portage Ave., Winnipeg, MB R3B 2E9, Canada; Department of Biology, The University of Winnipeg, 515 Portage Ave., Winnipeg, MB R3B 2E9, Canada; Department of Natural Resources and Northern Development, Province of Manitoba, Lac du Bonnet, MB R0E 1A0, Canada; Department of Wildlife, Fish, and Conservation Biology, University of California Davis, 1 Shields Ave., Davis, CA 95618, USA; Department of Animal Science, University of California Davis, 1 Shields Ave., Davis, CA 95618, USA; Cheung Consultancy Limited, 170 Forfar St, Maryhill, Dunedin 9011, New Zealand; NOAA Fisheries Southwest Fisheries Science Center, Fisheries Ecology Division and University of California Davis, Center for Watershed Sciences, 1 Shields Ave, Davis, CA 95618, USA; Indian Cultural Organization, 14840 Bear Mountain Rd, Redding, CA 96003, USA; Indian Cultural Organization, 14840 Bear Mountain Rd, Redding, CA 96003, USA; Indian Cultural Organization, 14840 Bear Mountain Rd, Redding, CA 96003, USA; School of the Environment, The University of Queensland, Brisbane, QLD 4072, Australia; Australia Zoo, 1638 Steve Irwin Way, Beerwah, QLD 4519, Australia; Australia Zoo, 1638 Steve Irwin Way, Beerwah, QLD 4519, Australia; Department of Biodiversity Conservation and Attractions, Kings Park Science, 2 Kattidj Close, Kings Park, WA 6005, Australia; School of Biological Sciences, University of Western Australia, Nedlands, WA 6009, Australia; Department of Biodiversity Conservation and Attractions, Kings Park Science, 2 Kattidj Close, Kings Park, WA 6005, Australia; School of Biological Sciences, University of Western Australia, Nedlands, WA 6009, Australia; Pilbara Environment and Cultural Knowledge Team, Rio Tinto, Central Park, 152-158 St Georges Terrace, Perth, WA 6000, Australia; School of Molecular and Life Sciences, Curtin University, Kent Street, Bentley, WA 6102, Australia; School of Biological Sciences, Adelaide University, North Terrace, Adelaide, South Australia 5000, Australia; Department of Biodiversity, Conservation and Attractions, Kings Park Science, 1 Kattidj Close, Kings Park, WA 6005, Australia; Ecotoxicology and Wildlife Health Division, Science and Technology Branch, Environment and Climate Change Canada, National Wildlife Research Centre, 1125 Colonel By Drive, Ottawa, ON K1S 5B6, Canada; Ecotoxicology and Wildlife Health Division, Science and Technology Branch, Environment and Climate Change Canada, National Wildlife Research Centre, 1125 Colonel By Drive, Ottawa, ON K1S 5B6, Canada; Ecotoxicology and Wildlife Health Division, Science and Technology Branch, Environment and Climate Change Canada, National Wildlife Research Centre, 1125 Colonel By Drive, Ottawa, ON K1S 5B6, Canada; Biology Department, Carleton University, 1125 Colonel By Drive, Ottawa, ON K1S 5B6, Canada; Nunatsiavut Government, Nunatsiavut Research Centre, P.O. Box 70, 12 Sandbanks Road, Nain, Labrador A0P 1L0, Canada; Nunatsiavut Government, Nunatsiavut Research Centre, P.O. Box 70, 12 Sandbanks Road, Nain, Labrador A0P 1L0, Canada; Department of Paraclinical Sciences and Centre for Veterinary Wildlife Research, Faculty of Veterinary Science, University of Pretoria, Soutpan Road, Private Bag X04, Onderstepoort 0110, South Africa; Veterinary Wildlife Services, South African National Parks, Kruger National Park, Private Bag X402, Skukuza 1350, South Africa; Marine Biology and Aquaculture, College of Science and Engineering, James Cook University, 1 James Cook Drive, Townsville, QLD 4811 Australia; Department of Biology and Institute of Environmental and Interdisciplinary Science, Carleton University, 1125 Colonel By Dr., Ottawa, ON K1S 5B6, Canada; Brain Function Research Group, School of Physiology, Faculty of Health Sciences, University of the Witwatersrand, 7 Yord Rd, Parktown 2193, South Africa

**Keywords:** Case study, collaboration, conservation physiology, co-production, government, Indigenous knowledge, management

## Abstract

As a relatively nascent discipline, conservation physiology has struggled to deliver science that is relevant to decision-makers or directly useful to practitioners. A growing body of literature has revealed that co-produced research is more likely to generate knowledge that is not only relevant, but that is also embraced and actionable. Co-production broadly involves conducting research collaboratively, inclusively, and in a respectful and engaged manner—spanning all stages from identifying research needs to study design, data collection, interpretation and application. This approach aims to create actionable science and deliver meaningful benefits to all partners involved. Knowledge can be co-produced with practitioners/managers working for regulators or stewardship bodies, Indigenous communities and governments, industry (e.g. fishers, foresters, farmers) and other relevant actors. Using diverse case studies spanning issues, taxa and regions from around the globe, we explore examples of co-produced research related to conservation physiology. In doing so, we highlight benefits and challenges while also identifying lessons for others considering such an approach. Although co-production cannot guarantee the ultimate success of a project, for applied research (such as what conservation physiology purports to deliver), embracing co-production is increasingly regarded as the single-most important approach for generating actionable science to inform conservation. In that sense, the conservation physiology community would be more impactful and relevant if it became commonplace to embrace co-production as demonstrated by the case studies presented here.

## Introduction

A long-standing challenge in protecting and restoring biodiversity is connecting science to decision-makers to ensure that conservation and management policies and actions are evidence-based (Sutherland *et al.,* 2004). This has been variously termed the knowledge-action gap, science-policy gap and theory-practice gap, among others (Cooke *et al.,* 2020), and is pervasive in conservation (Bradshaw and Borchers, 2000; Bertuol-Garcia *et al.,* 2018). The reasons for this gap are numerous, with responsibility held jointly by knowledge generators and knowledge users. Although individual-level traits and actions (or inactions) contribute to the gap, so do significant institutional and cultural constraints that reinforce or even broaden the gap. In a critical and urgent discipline like conservation, such gaps are problematic and must be narrowed or bridged for the benefit of biodiversity and people.

Although there are many efforts that can and should be undertaken to narrow or bridge the gap (e.g. institutional reform, open science, use of knowledge brokers), one of the most practical efforts that can be embraced by knowledge generators and users with relative ease is co-production. Co-production can take many forms but generally involves conducting research collaboratively, inclusively, and in a respectful and engaged manner—from the identification of research needs to study design, data collection, interpretation and even application—with the idea of creating actionable science and benefits to the partners involved (Norström *et al.,* 2020). Knowledge can be co-produced with practitioners/managers, Indigenous communities/governments, industry (e.g. fishers, foresters, farmers) and other relevant actors. Importantly, co-production involves considering evidence in real-world contexts that are relevant to society and decision-makers (van der Hel, 2016) thus inherently invoking concepts such as social justice and equity and providing voices and agency for marginalized groups that are often impacted by conservation decisions (Moallemi *et al.,* 2023; Morris *et al.,* 2024). Not only is co-production practical and accessible to individuals, but it has also been demonstrated to yield outcomes for conservation and people that are considered beneficial (Miller and Wyborn, 2020). To that end, there are now resources available to support those wishing to engage in co-production (see Beier *et al.,* 2017; Norström *et al.,* 2020). Yet, co-production in science is still not the default method and is often dependent on interpersonal relationships that take substantial time and investment to develop (Arnott *et al.,* 2020; Cooke *et al.,* 2021a). Therefore, it is important to share relevant case studies to learn from successes and failures.

Conservation physiology is a relatively nascent discipline focused on the application of physiological knowledge, concepts and tools to understand and solve conservation problems (Tracy *et al.,* 2006; Wikelski and Cooke, 2006; Cooke *et al.,* 2013). Although there are a growing number of success stories where conservation outcomes have benefited from physiology (reviewed in Madliger *et al.,* 2016), there are also many inherent challenges that make it difficult for physiology-based research to be embraced and used by decision-makers (Cooke and O’Connor, 2010). Examples include physiologists being unaware of how conservation decisions are made, while conservation practitioners often are unaware of the potential use of physiology, physiological research done in laboratory settings lacking direct ecological relevance, a reliance on model or surrogate species, use of invasive methods and an inability to scale physiological endpoints to ones of relevance to decision-makers (i.e. fitness, population dynamics, community structure and function; reviewed in Cooke and O’Connor, 2010; Bergman *et al.,* 2019; Ames *et al.,* 2020; Madliger *et al.,* 2021a, 2021b). Despite these challenges, conservation physiology has significant potential to inform decision-making if it is effectively integrated with co-production approaches that foster collaboration between scientists and stakeholders. Laubenstein and Rummer (2020) argue that one of the key limitations of conservation physiology is the communication gap between physiological researchers and applied conservation efforts. Conservation physiology is often perceived as highly technical, requiring specialized expertise, which can make it inaccessible to decision-makers. However, when conservation physiology is co-produced with practitioners, Indigenous groups or policymakers, physiological insights can be directly translated into conservation strategies, as demonstrated in co-produced projects focusing on thermal tolerance and stress physiology of Pacific salmonids (see Cooke *et al.,* 2012; Hinch *et al.,* 2012; Cooke *et al.,* 2021b). Such examples highlight how physiological research can be reframed to address management-relevant questions, increasing the likelihood of real-world conservation impact. The concept of co-production would therefore seem to be an obvious and effective means of overcoming or addressing some of those issues specific to conservation physiology and that reinforce the knowledge-action gap. Indeed, the aforementioned cases on research in Pacific salmon (Cooke *et al.,* 2012; Hinch *et al.,* 2012; Cooke *et al.,* 2021b) specifically noted the important role of co-production in achieving impact on policy and practice. Yet, although there have been efforts to reframe conservation physiology to be more impactful, inclusive and equitable, an explicit call for co-production has not been made (Cooke *et al.,* 2020).

Here, we explore the concept of co-production and its relevance to conservation physiology and the opportunity to improve conservation outcomes for biodiversity and people. To do so, we assembled a diverse suite of case studies that embraced co-production in conservation physiology projects and that span region, taxa, issue and partners. Themes related to benefits, challenges and lessons were extracted from case studies to identify commonalities. If co-production were more fully embraced by the conservation physiology community, we posit that the discipline would quickly become even more relevant and in doing so would increase its role in generating actionable evidence to inform conservation and management decisions.

## Case Studies

Case studies were identified by asking members of the editorial board of the journal *Conservation Physiology* to identify potential authors or projects (including self ‘nomination’) where co-production had been embraced. In addition, invitations were extended via X (formerly, Twitter) in July of 2024 to the broader community of conservation physiologists and the tweet was shared by the Conservation Physiology journal twitter account. Unfortunately, the efforts to share on social media failed to yield additional cases such that all of those presented are in some way connected to members of the editorial board. After expressions of interest were made by potential contributors, a formal invitation was extended that included an overview of the goal of the paper, details of expectations for co-authorship and a summary of the information required from contributors (i.e. a ~ 250 word case study and a summary of key benefits, challenges and lessons related to embracing co-production in conservation physiology projects). Here, we present the case studies (see Table 1) followed by a summary of those key benefits, challenges and lessons learned; many of these span the different case studies. Although we attempted to include examples including all taxa and regions, there was a concentration of cases from North America (with a single case study from each of Africa and Australia) dominated by examples involving vertebrates (only one plant example) and, more specifically, cases on salmonid fishes. Salmonids are ecologically, economically and culturally valuable such that they are the subject of intensive management as well as stewardship by Indigenous peoples. Co-production is a ‘natural’ fit with salmonid research. We acknowledge that each case study is somewhat different in terms of structure and level of detail. Each case study was co-produced (co-written) by team members from a given project, and it was important to ensure that each one was able to tell their story in their own voice given we have diverse positionality (e.g. from Indigenous leaders to government regulators to NGO representatives).

**Table 1:** Summary of the characteristics of the case studies on conservation physiology presented in this paper

**Case study**	**Location**	**Key partners**	**Biomarkers**
Mitigating the impacts of hydropower on Pacific Salmon	Seton River, British Columbia, Canada	Academic researchers, Indigenous rightsholders and resource managers, hydropower utility practitioners, government scientists and regulators	Bioenergetics, swimming ability (including passage success), vitality (reflex impairment), survival
Clinical management of immobilized white rhinoceros	National Parks in southern South Africa	Academic researchers, government veterinary professionals and scientists, park managers	Health, metabolic function, cardiopulmonary function
Defining plant-niche impacts to guide flora conservation decisions	Western Australia	Academic researchers, resource managers, government scientists and regulators	Growth, respiration, environmental tolerances, survival
Health research for Wood Bison	Alberta, Canada	Government scientists, Indigenous knowledge holders, recovery planning team	Glucocorticoids, contaminants, health, survival
A collaborative approach to an emergency diesel spill response	Kaipokok Bay, Nunatsiavut in Newfoundland and Labrador, Canada	Government scientists and regulators, academic researchers, Indigenous government leaders	Methylation levels of DNA, gene expression, endocrinology, population persistence
Honouring Indigenous knowledge to rematriate Nur (salmon) to Northern California’s Winnemem Waywaket (McCloud River)	New Zealand and California (Winnemem Waywaket—McCloud River)	Indigenous leaders, scientists and resource managers, academic researchers, farmers, social justice advocates	Swimming performance, growth, survival
Understanding the consequences of recreational angling on Lake Trout in Manitoba	Clearwater Lake in Manitoba, Canada	Academic researchers, government managers, regulators and scientists, recreational fishing industry (guides and lodges)	Swimming ability, survival, glucocorticoids, blood physiology, egg viability/performance
Crocodile conservation through scientific discovery, outreach and education	Coastal and estuarine waters of northern Australia	Academic researchers, zoo scientists and practitioners, government resource managers	Space use and habitat selection, movement, thermal biology, survival

### Mitigating the impacts of hydropower on Pacific Salmon

The Seton Dam, approximately 350 km upstream from the Fraser River estuary in the southwestern British Columbia, Canada, is situated near the end of the migratory route for a population of Fraser River sockeye salmon (*Oncorhynchus nerka*). The impacts of dams on migratory fish physiology are complex and can be difficult to manage (Hinch *et al.,* 2022), but in this scenario, they were successfully addressed through a collaborative effort by resource managers, scientists and engineers working with and for First Nations governments, industry, parliamentary government and academic institutions (Bett *et al.,* 2022). The researchers worked together to define their research aims, design experiments, collect data and apply the results to the management of the dam. A series of streamside and *in situ* experiments identified how to divert water to the facility’s power station while allowing migrating salmon to navigate through the region, and how to release water through the dam’s siphons and fishway while reducing adverse physiological consequences of passage (assessed using electronic tags with acceleration sensors; Burnett *et al.,* 2014) and promoting survival to spawning grounds. Physiological knowledge enabled the team to identify specific mechanistic relationships between dam operations and fish condition, but that work extended to include endpoints of primary interest to the regulator and First Nations peoples (i.e. survival to spawning grounds). The project’s findings and, critically, their application to salmon conservation could only be achieved through the collaborative involvement of the crown corporation that operates the dam (i.e. BC Hydro), the First Nations rightsholders of the territory, the technicians who conducted the experiments (some of whom were from the local First Nation), and the specialists who interpreted the data in the context of scientific findings as well as Indigenous knowledge systems. The end result was a positive one: operations of the dam were adapted in accordance with study findings, providing improved conditions for salmon that promote not only successful navigation and dam passage, but also post-passage survival to spawning grounds. Findings were rapidly embraced because they were co-produced, such that there was collective support for and trust of the process and resultant findings by all parties involved.

### Clinical management of immobilized white rhinoceros (*Ceratotherium simum*)

The white rhinoceros (*C. simum*), one of five extant species of rhinoceros, is threatened by poaching and habitat loss. As the main custodian of rhinoceros in southern Africa, South African National Parks plays a major role in the conservation of this species. Their conservation approach is hands-on as it requires frequent management interventions, like dehorning and animal translocations, to mitigate the threat of poaching and maintain the health of rhinoceros populations. These interventions require that rhinoceros are captured and can be handled, which is achieved by darting animals using reversible immobilizing drugs. Although these drugs are effective in immobilizing rhinoceros, they are highly potent and cause adverse physiological effects that pose a risk to health and survival. The extent of the adverse effects and the underlying mechanisms are not completely known, making it difficult to develop effective interventions to treat or prevent adverse consequences. Researchers with expertise in physiology and pharmacology worked together with South African National Parks Veterinary Wildlife Services to develop projects to address these problems. Co-production of these projects involved jointly defining research aims, designing the studies, collecting data and assessing and publishing results. Key to success was the capture and temporary housing of rhinoceros in purpose-built holding facilities, a task requiring specific expertise and husbandry (Miller *et al.,* 2022) from dedicated Veterinary Wildlife Services staff. Through co-produced research with VWS’s veterinarians, the researchers developed novel techniques to extensively measure cardiopulmonary and metabolic function, not a trivial task considering the massive size of the rhinoceros used (1.2–1.6 tonnes). Pharmacological and physiological knowledge enabled the team to quickly establish interventions that improved physiological welfare and effectively reduced morbidity and mortality risks for the rhinoceros during immobilization (Haw *et al.,* 2014; Buss *et al.,* 2018; Buss *et al.,* 2024). Additionally, through a series of projects that provided further insights into the mechanisms underlying the adverse effects (Buss *et al.,* 2016; Buss *et al.,* 2017; Boesch *et al.,* 2018; Mosing *et al.,* 2020), the research team was able to refine these interventions. Altogether, the result has been positive as it permitted the immediate use of risk-reducing interventions (Haw *et al.,* 2015) and allowed South African National Parks’ veterinarians to make more informed in-field clinical decisions when capturing rhinoceros. Based on the credibility of the institutions involved, outcomes were also rapidly embraced by the broader wildlife veterinary profession with a reduction in mortalities associated with chemical capture in white rhinoceros.

### Defining plant-niche impacts to guide flora conservation decisions

Banded ironstone formations in Western Australia have substantial biodiversity value, characterized by high species endemism, where many species are restricted to individual outcrops (Byrne, 2019). At the same time, the ironstone is some of the highest quality iron ore in the world (Courtney-Davies *et al.,* 2024), with the iron ore mined in Australia’s northwest representing 38% of global exports in 2022 (Government of Western Australia, 2023). High-quality conservation decisions need to be underpinned by critical biological knowledge (Stewart *et al.,* 2005; Gillson *et al.,* 2019), particularly when range-restricted species and mineral resource development intersect. A multidisciplinary research program involving themes of ecology, ecophysiology and biogeography (Lewandrowski *et al.,* 2024) brought together expertise from resource managers, conservation biologists and scientists to quantify interactions between plants and their environment to understand the persistence of *Aluta quadrata* (Rye & Trudgen), a threatened plant species from the northwest arid region of Western Australia. Biogeographical modelling estimated graduated habitat suitability, both within the known extent of the species, and outside of it, to identify populations that may be differentially vulnerable to environmental change. Physiological assessments of these populations found that plant performance was highest in high suitability habitat during wet seasons, but climatic stress impacted all populations equally. Therefore, population persistence may be driven primarily by performance during wet seasons, rather than resilience through the dry. Typically, mine planning is guided by legislative requirements around initially avoiding environmental impacts, and where unavoidable, minimizing those to remnant populations. The high degree of engagement between research institutions, resource managers and regulators early in the process of mine planning facilitated a holistic understanding of the patterns and processes underpinning the persistence of the species, identifying the habitat critical to the species. This degree of engagement has facilitated conservation management of the species to transcend typical monitoring conducted under legislative requirement. While *A. quadrata* populations have not yet experienced substantial direct disturbance through mining activity, the outlook for planning any future activity looks positive, largely because of the co-production approach to species management that began early in the process.

### Health research for wood bison (*Bison bison athabascae*)

Wood Bison (*B. bison athabascae*) are an iconic species in the northern Canada and play a critical role in maintaining ecosystem dynamics, such as influencing vegetation structure and nutrient cycling. Despite their extensive range, the maintenance and recovery of specific bison populations has faced significant threats (Environment and Climate Change Canada, 2020a). In 2018, through a partnership between federal, provincial/territorial and Indigenous partners, a recovery strategy for Wood Bison in Canada (Environment and Climate Change Canada, 2018) was developed that identified key objectives for the recovery of this ‘threatened’ species (Government of Canada, 2020). Environment and Climate Change Canada subsequently released the Imminent Threat Assessment for Wood Bison (Environment and Climate Change Canada, 2020a) and an Imminent Threat Order was declared by the Minister of Environment and Climate Change Canada, noting that conservation threats to the Ronald Lake and Wabasca Wood Bison herds could, in the near-term, affect recovery objectives (Environment and Climate Change Canada, 2020b). While there exists a range of conservation threats to Wood Bison, three principal threats were identified that require immediate research and management actions. For the Ronald Lake Bison herd, these include threats from infectious diseases introduced from domestic cattle (Environment and Climate Change Canada, 2018), notably bovine tuberculosis (*Mycobacterium bovis*) and brucellosis (*Brucella abortus*), and habitat loss and degradation. For the 20 individual Wabasca Bison herd, unregulated harvest is also a key concern (Environment and Climate Change Canada, 2018, 2020a, 2020b). Additionally, these herds are vulnerable to stochastic events, like anthrax outbreaks and extreme weather, which can cause significant population fluctuations (Environment and Climate Change Canada, 2020a). In response to the Ministerial Order, federal researchers in Environment and Climate Change Canada’s Science and Technology Branch developed a research plan to examine Wood Bison health and physiological responses to key conservation threats to provide support for management actions and the recovery of the Ronald Lake and Wabasca Bison herds. Glucocorticoid level, specifically cortisol, is a well-established biomarker of long-term stress in mammals (Macbeth *et al.,* 2010) and prolonged elevated glucocorticoid levels can negatively impact survival and fitness (Mooring *et al.,* 2006). Active validation of cortisol measurement techniques is currently underway for bison, with the goal of analyzing non-invasively collected hair samples. The methodology is intended to provide species-specific insights into stress and its potential impacts on survival and fitness. Further incorporation of environmental and demographic variables, as well as comparison across herds and to historical samples, is planned to provide insight into the impact of environmental stressors on Bison. Additionally, a project is underway to engage with Western-trained management leads to identify sampling approaches and diagnostics to characterize aspects of Bison health and disease. Ongoing research is assessing contaminant levels, notably trace elements, in Wood Bison hair and faeces, their forage, and habitat (e.g. soil, water), providing up-to-date data that can be compared to historical data dating back to the 1970s (Wilcox *et al.,* 2023). Landscape-level modelling will show the spatial distribution of trace element concentrations, while climate change projections will be integrated to address changing habitat conditions and potential future northward migration of the herds. Combined, the research plan lays out proposed approaches to establish physiological drivers of Wood Bison health and collaboratively conduct research with governmental and non-governmental partners invested in the maintenance and recovery of the at-risk herds.

### A collaborative approach to an emergency diesel spill response

In June 2020, Inuit community members detected a diesel oil spill in Kaipokok Bay near Postville, Nunatsiavut in Newfoundland and Labrador on the east coast of Canada (Fletcher *et al.,* 2023). This spill led to a suite of emergency response actions by the several federal government groups that are tasked with understanding the impacts of spills on the environment and cleaning up spills, when applicable (Fletcher *et al.,* 2023). The Nunatsiavut Government was not only concerned with the immediate lethal impacts from the spill, but also concerned with the longer term, more subtle sub-lethal impacts on migratory birds, because the spill occurred as migratory birds were starting to breed in the area (Zahaby *et al.,* 2021; Sarma *et al.,* 2022). Breeding bird species in the area included some that are commonly harvested as part of the subsistence hunt. Based on previous relationships between the Nunatsiavut Government and federal and academic researchers, a research plan was developed in the days after the oil spill that aimed to look at the chemicals the birds were exposed to, and the effects these chemicals may have on several physiological metrics, including methylation levels of DNA, gene expression and hormone production (Ho *et al.,* 2025; Zahaby *et al.,* 2025). The research group (Nunatsiavut Government, federal scientists and academics) co-developed a plan on which species to focus on, when to collect samples, what analyses should be prioritized, and how to collect, process and ship the needed samples for the desired analyses (Provencher, Unpublished Data). All members of the group contributed ideas to what could be done, with the Nunatsiavut Government having the final approval of the research plan. Federal scientists and academics, along with students, received the samples from Nunatsiavut Government partners, who collected the samples on the ground. The research laboratories carried out the analyses, data handling and processing, and brought data summaries to the larger collaborative group to discuss and co-analyze together. This approach made it possible to apply biological context to physiological data, and for partners to offer insights into why physiological metrics may differ between species or groups based on behaviour and ecology of the species in the region where collections were carried out. As coastal Indigenous communities have disproportionate risks from increasing marine shipping and oil development due to their harvest and consumption of marine birds, the use of physiological tools to understand the implications for oil spills is critical to a holistic understanding of impacts on the environment.

### Honouring Indigenous knowledge to rematriate Nur (Salmon) to northern California’s Winnemem Waywaket (McCloud River)

The Winnemem Wintu are the ‘middle water people’ who inhabit ancestral territory from Bulliyum Puyuuk (Mt. Shasta) down the Winnemem Waywaket (McCloud River) watershed in Northern California, USA. The Winnemem Waywaket was once known as the best salmon (Nur; *Oncorhynchus tshawytscha*) breeding river in the world (California State Board of Fish Commissioners, 1890; Yoshiyama *et al.,* 2001) and Winnemem Wintu Tribal oral histories also attest to large numbers of Nur pre-European contact, filling the rivers so full of fish that one could walk over them. In contrast, Chinook salmon runs in California including the endangered Sacramento River Winter-Run, threatened Central Valley Spring-Run and Late Fall/Fall-Run are each currently facing increasing risks of extinction, and the second year of a closed fishery for the latter (Johnson *et al.,* 2023). When the Shasta Dam was constructed during World War II, it flooded the Winnemem homelands and blocked the salmon runs from accessing cold-water spawning streams originating from Bulliyum Puyuuk. In the late 1800’s, Winnemem Waywaket Chinook salmon eggs were also stolen from their homeland and shipped to New Zealand to start a fishery on the South Island. Through the vision and leadership of Chief Caleen Sisk, the Winnemem Wintu Tribe has catalyzed the formation of an unlikely and influential group of people, including farmers, scientists, environmentalists and social justice advocates, who are united in the spirit of reconciliation to restore the Winnemem Waywaket as California’s cold-water stronghold for Nur. The scope of the effort is large, including elements of volitional passage, fish rearing and reintroduction, and large-scale habitat restoration, with all weaving together Indigenous and Western sciences. Indigenous science has a deep history rooted in holistically promoting animal health through ecosystem health, a fundamental approach that has not been fully realized in present-day fish conservation measures. One particularly unique element to this effort is the Chief’s vision for how Nur will be reintroduced to the Winnemem Waywaket and her insistence that all methodologies are designed to promote ‘wildness’, avoiding traditional Western science hatchery practices such as the use of heath trays for rearing fry. To this end, Chief Sisk designed the Nature-based Nur Incubation System, which was successfully constructed by UC Davis Fish Conservation Physiology Labs to reintroduce endangered SRW Nur into Winnemem Waywaket. The Nur Nature-based Incubation System uses Winnemem Wintu Indigenous knowledge to better prepare Nur for life in the river and their marathon swim out to the ocean before returning approximately 3 years later to spawn. Winnemem Wintu Indigenous science indicates that fish reared in a more natural setting, including natal water flows, natural food, rocks, plants and intrinsic medicines, will promote the characteristic traits needed for Nur to regain their abilities to be mountain climbers. This is important so that they can once again ascend the high-elevation habitat at the base of Bulliyum Puyuuk when they return to their ancestral waters to spawn. The Nur Nature-based Incubation System offers a window to observe natural behaviours in the early life stages of development of Nur, such as swimming, predator avoidance and natural foraging, allows fish to volitionally choose when to enter the Waywaket, and gives tribal cultural resource specialists/researchers the ability to track growth and survival non-invasively. This system has been successfully used to rear Sacramento River Winter-Run Nur on the Winnemem Waywaket for two consecutive seasons. The co-production of knowledge gleaned through the development of the Nur Nature-based Incubation System will ultimately lead to a sustainable fishery for the Winnemem Wintu people and the restoration of all Chinook salmon runs including the rematriation of Winnemem Nur from New Zealand that were stolen from Winnemen Waywaket over a hundred years ago. With Shasta Dam blocking access to Nur’s ancestral habitat and droughts projected to intensify in California’s future, returning these salmon to their cold-water refuge—where they can be stewarded again by the Winnemem Wintu—is essential for ensuring their survival and enhancing climate resilience.

### Understanding the consequences of recreational angling on Lake trout in Manitoba

Clearwater Lake in the northwestern Manitoba, Canada is a clear oligotrophic lake, prized by recreational anglers from across the world for its trophy lake trout (*Salvelinus namaycush*). Anglers practice catch-and-release angling, with the assumption that released fish display negligible impairment. However, the impacts of angling on large lake trout are poorly understood, and additional information is needed to ensure the sustainability of the fishery. To address this lack of knowledge and to inform decision-making, Manitoba’s provincial Fisheries Branch and the University of Winnipeg (Fish Biology and Conservation Laboratory) collaborated to co-produce several original research projects on lake trout. Regional fisheries managers, biologists and scientists took part in every step of the process, including conceptualization of projects, investigation and subsequent publication of data; and local stakeholders such as guides, and lodge owners participated in data collection and were kept informed as the studies progressed. Several *in situ* experiments identified how angling of lake trout influenced percent mortality, reflex impairment, extent of barotrauma, physiological status (i.e. blood cortisol, lactate, glucose, pH) and post-release locomotor activity (DePasquale *et al.,* 2023; Howell *et al.,* 2023; Howell *et al.,* 2024). Results indicate that while lake trout may survive catch-and-release angling, factors such as fight time, air exposure, angling depth, sex and season can increase sublethal impairments. Catch-and-release angling will continue; however, care should be taken to minimize stressors by adhering to best practices for fish handling. Ongoing research aims to explore the effects of angling on spawning lake trout and how maternal stress may impact the developing eggs and offspring (Schoen, Unpublished Data). Eggs will be tested for mortality, stress hormones, energy content and genetic markers of stress. Together, these studies form comprehensive analyses on the impacts of recreational angling on lake trout. These studies are the first in several decades to assess lake trout in Manitoba, specifically, providing contemporary data and analysis, which are critical in protecting and developing fisheries. The success of these integrative projects, and their potential to inform policy, is only possible through the combined effort of Manitoba’s provincial Fisheries Branch, researchers from the University of Winnipeg, volunteers during the field programmes, and the assistance of local fishing lodges and guides. This work will lead to several published research articles, knowledge sharing with local angling groups and the potential for regulatory changes.

### Crocodile conservation through scientific discovery, outreach and education

The estuarine crocodile *Crocodylus porosus*, the world’s largest species of crocodilian, suffered severe population declines in Australia from hunting before being protected in the 1970s (Webb *et al.,* 2010). Recovery has been highly variable across Australia and threats remain including illegal hunting, exploitation, fishing by-catches, loss of habitat and the predation of eggs by feral pigs. Climate warming and increases in extreme weather events are also predicted to impact upon *C. porosus* in the future (Elsworth *et al.,* 2003; Rodgers and Franklin, 2017, 2019; Barham *et al.,* 2024). A long-term partnership and collaboration between researchers from the University of Queensland and conservationists and crocodile experts from Australia Zoo, Queensland have been focussed on the protection and conservation of *C. porosus*. A large part of this team’s conservation action strategy has been through education and public dissemination of the scientific discoveries via social media and TV but also through lobbying policy makers. This research team has pioneered the use of archival tags, acoustic and satellite telemetry to monitor crocodilians (Franklin *et al.,* 2009). In 2008, the research team embarked on an ambitious long-term acoustic and satellite telemetry study to monitor the movements, behaviours and physiology of crocodiles in the face of future climate change (Barham *et al.,* 2025). For 17 years, a team of 15–20 personnel has travelled to the Steve Irwin Wildlife Reserve, Cape York Peninsula, Queensland to conduct research on the Wenlock River—capturing estuarine crocodiles, measuring them, taking blood and tissue samples, and implanting and attaching transmitters. To date more than 270 animals, ranging in body length from 1 to 4.65 m, have been captured and more than 8 million individual recordings of body temperature have been recorded since 2008. Coupled with measurements of body temperature are movement and diving data to determine the potential impacts of changes in body temperature on performance with climate warming (Barham *et al.,* 2025). This study represents the largest and longest running tracking study of its kind for any species of crocodilian and there is an on-going commitment to continue for at least the next 10 years (the lifespan of acoustic tags implanted in 2024). This collaboration has generated a substantial body of research that has advanced our understanding of the behaviour, movement ecology and physiology of estuarine crocodiles. Research findings have been used to promote awareness of the importance of apex predators in ecological processes (thus building public support for their protection), to manage problem crocodiles and to prevent human–crocodile conflict.

#### Benefits

As demonstrated by a growing body of literature that extends from health care to conservation, and the case studies we describe, there are numerous benefits derived from embracing co-production. Specific to conservation physiology, we identified the following benefits of embracing co-production:

##### Generates relevant and actionable knowledge to inform decisions

Co-produced research allows researchers to design studies that directly address management needs and policy questions. That means that research is fit for purpose and directly relevant to end users such that it can contribute to bridging the science-action gap (Arlettaz *et al.,* 2010; Bertuol-Garcia *et al.,* 2018). Indeed, co-production is often identified as the single most important way to generate relevant and actionable knowledge. Nearly, all of the case studies presented here embraced co-production to do just that.

##### Builds trust among partners

Trust is fundamental to building consensus and creating an environment where new knowledge is valued and embraced. If trust is absent, then even knowledge that may otherwise be robust may be ignored. Developing trust requires time and should begin early as relationships are established during the initial stages of co-production, as relationships develop, and partners align on goals. However, trust is fragile. It takes time to earn but can be quickly lost if not actively maintained. By engaging stakeholders and rightsholders early and maintaining continuous collaboration, co-production fosters trust and ensures that conservation physiology research is directly applicable to management decisions (Laubenstein and Rummer, 2020). This trust is particularly important in projects involving Indigenous knowledge and science, as seen in the case study on salmon rematriation and in the diesel spill case, where Indigenous concerns shaped research methodologies. Such partnerships allow for reciprocal knowledge exchange and ensure that research outcomes are co-owned and embraced by all stakeholders.

##### Strengthens relationships and creates opportunities to learn from each other in the long-term aiding future conservation efforts

Co-production can be an iterative process that leads to the next set of questions of importance to the group. This was particularly apparent in the case study (above) arising from the oil spill in Nunatsiavut in eastern Canada where the initial learning extended over years while the project adapted and expanded as new knowledge and understanding was achieved by the group. Moreover, the relationships developed through co-production do not end with individual projects. Following the completion of projects, subsequent collaborative studies often take place. A partnership between groups opens the door for sustainable, long-term research that is not reliant on the involvement of a small number of key individuals. Such long-term projects are sorely needed in conservation physiology, as they will allow for management decisions that enable adaption to future environmental change.

##### Supports the upholding of Indigenous rights and data sovereignty

Conservation research often occurs on traditional, ancestral and unceded territories of Indigenous peoples. Local Indigenous governments and communities, however, are regularly excluded from research as well as ownership of data collected on their lands. Co-production is inherently positioned to enable respectful collaboration with Indigenous peoples (i.e. addressing the oft-stated Indigenous assertion ‘nothing about us without us’ as per the UN Declaration on the Rights of Indigenous Peoples), while upholding Indigenous data sovereignty by directly involving Indigenous peoples in the research from the early phases, by providing opportunities to co-develop protocols for data collection, use and storage (e.g. the CARE principles for Indigenous Data Governance; https://www.gida-global.org/care). Moreover, in some cases where Indigenous knowledge is braided, bridged or woven with western science a two-eyed seeing approach (see Reid *et al.,* 2021) can be used to ensure that Indigenous knowledge is not co-opted and subsumed in a colonial manner. Several of the case studies presented here involved addressing issues related to data sovereignty prior to fully partnering on research projects.

##### Fosters cross-disciplinary studies

The exploratory nature of cross-disciplinary studies can make them difficult to justify in conventional, competitive research funding applications. It is often difficult to determine what the outcomes will be of blending different measurement techniques, data sets and analytical paradigms or indeed whether integration is even possible. Co-production provides a framework to navigate these uncertainties by allowing focused, outcome-driven collaboration within a defined project scope.

##### Enables one to bring their toolbox to a wider audience

The development of new approaches can be beneficial beyond the immediate scope of a study. Whether reimagining research frameworks that have benefits to the wider field of conservation (Stephen *et al.,* 2023) or developing new techniques to obtain physiological metrics from non-invasive samples (e.g. faeces, hair, skin), a project can become a case study of a novel approach. In doing so, the removal of disciplinary silos encourages innovation and progress that might not occur if findings were not co-produced.

##### Builds long-term research and conservation capacity

Long-term partnerships (e.g. 17 years in the case of the crocodile case study) enable the development of substantial research infrastructure, expertise, methodology and datasets that benefit both current and future conservation efforts that extend beyond focal taxa. Long-term relationships enable the development of comprehensive datasets that provide unique insights into fundamental and applied aspects of organismal biology with strength in identifying responses to environmental change.

##### Creates legacy

Most research projects have a pre-defined future. For a truly long-term research (and relationships with partners), plans need to be made for the project potentially outlasting those directly involved. Working with a government, tribal or industry partner gives the science potential for generational research. Strategies may include a ten-year operational plan, building capacity within communities, budgeting for continuing research and successional planning. The longer a research project can continue with consistency and support, the greater the long-term outcomes.

##### Enables potential for doing management-scale experimentation

Research done in the conservation physiology space is notoriously done at scales that make it challenging for managers to be confident that findings will be relevant at a management scale. Co-produced studies that are done at a management scale can bring confidence to decision-makers and relevant parties. Such tests require buy-in and collaboration from a variety of groups. The case study on salmon–dam interactions involved a collaborative partnership between the dam operators, rightsholders, managers and scientists. The findings obtained from the *in situ* experiments provided evidence that was critical to the implementation of solutions to the proposed management changes.

##### Develops key skills

Collaboration fosters personal and professional growth and strengthens expertise. In particular, trainees that take part in co-produced research learn about different knowledge systems, the importance of relationships in conservation, how to integrate physiology into impactful conservation actions and generate knowledge that is relevant to decision-makers. Those skills are in high demand by employers. As such, often those involved (i.e. students and post-doctoral researchers) are often hired by collaborators, as was the case in the lake trout study. Indigenous ranger programs provide important pathways for traditional owners to seek employment that maintains contact with the land (Wright *et al.,* 2021). Integrating physiological studies with such programs, while currently not common, can only elevate the technical skills acquired and applied in such settings.

##### Provides a safety network

Conservation physiology research that takes place in remote areas requires an incredible amount of planning and adaptability. Unexpected challenges such as extreme environmental temperatures, equipment malfunctions, resource scarcity or logistical issues can all pose roadblocks to research. Co-production allows researchers to establish support networks comprised of people who have access to local knowledge and resources. Ultimately, this approach improves project success and team safety.

##### Creates pathways for addressing human–wildlife conflict

Although many other benefits identified are somewhat generic, one specific area of conservation science and practice where co-production may be particularly useful is in identifying pathways for addressing human–wildlife conflict. In the context of the crocodile example, the partnership’s research has directly contributed to reducing human–wildlife conflict by developing quantitative methods to assess and communicate crocodile presence probabilities in specific locations and times (Campbell *et al.,* 2014). This data-driven approach enables evidence-based risk assessment that can inform public safety measures while supporting crocodile conservation. The combination of detailed behavioural research and public education has created more effective strategies for promoting human–crocodile coexistence, demonstrating how scientific research can directly contribute to conservation outcomes through improved community acceptance and reduced retaliatory killing.

##### Divides costs

The summary of these themes is ultimately that co-production decreases a host of costs that often limit conservation activity generally, and conservation physiology research specifically. Financially, such research is often too costly for universities, not-for-profit agencies or traditional communities to support alone, but too speculative or too specific for industry or governments to justify. Many government funding agencies offer industry-supported research grants that look well on genuine co-production between industry and research (such as the Australian Research Council Linkage Scheme; see https://www.arc.gov.au/funding-research/funding-schemes/linkage-program/linkage-projects) and increasingly seek to promote integration between western science and traditional ways of knowing. Genuinely co-produced research leverages the financial costs by defraying infrastructural and in-kind support, as well as cash investment, across groups. However, costs are more than just financial: co-production defrays the cost of institutional knowledge across partners as well. Data are generally stored in multiple places, with such redundancy insuring against loss or compromise at any one place. Similarly, the loss of single, key personnel is insured against by the relationships built. Finally, social licence is insured, especially for conservation physiologists.

#### Challenges

Although co-production can yield benefits (as outlined above), it also can face several challenges. Based on our collective experiences in engaging in co-production related to conservation physiology, some of the more notable challenges include:

##### The need to balance diverse opinions

Broadening a team of collaborators and partners inherently bolsters the research through increased scrutiny in all stages of the project. This translates to more discourse during identification of priorities, knowledge-sharing or even publication. Expectations and interests vary, yet meeting the needs of multiple partners is essential. Having conversations early-on about how goals of team members can be achieved concurrently will help avoid priority shifts in the later stages of the project. To be clear, one of the reasons to embrace co-production is for diverse perspectives to be included so this is not a criticism of the approach but simply a reality that more participants require more effort to achieve consensus. Having more partners also means more objectives and the potential need for negotiated solutions. Moreover, it should be noted that Indigenous communities are different from other partner groups and historically, some government agencies have not respected the sovereignty of Indigenous organizations. In some cases this has led to co-production being forced upon Indigenous communities by government agencies, rather than allowing Indigenous peoples and organizations to freely choose this process within their own right. Consequently, this can cause unwanted tension among parties and inherently goes against the nature of co-production. As such, successful co-production requires good intentions, the careful and collaborative consideration of different objectives, and assurance that they are met to a satisfactory level for all involved.

##### Concessions are necessary

Co-production will often require compromise between those involved in the research and a willingness for negotiated solutions. Co-production requires careful and active listening and a willingness to find a path that works best for all—that is the spirit of co-production. As such, it is likely that compromises occur all throughout the research process and not only at points of conflict. Moreover, during these negotiations, mutual respect and reciprocity must be had, to ensure meeting the highest priorities for individual parties. Often, this also comes with a willingness to concede one’s own preferences on lower priority actions to support the priorities of a partner.

##### Co-production done well takes time

Co-production cannot be rushed, and this can lead to mismatches in timelines. Community groups may have long-term commitments to an issue that span decades (or even generations for Indigenous communities), while industry partners often operate within shorter financial or regulatory cycles. These differences can create challenges in aligning expectations and deliverables. This highlights the importance of managing expectations within these partnerships regarding timelines. As such, each partner likely has different time constraints, and these should be communicated early in the planning process to ease research progress. Similarly, students involved in co-produced projects often face academic deadlines that do not always align with the slower, iterative nature of collaborative research. While integrating student research into co-production can provide valuable learning experiences and fresh perspectives, it requires careful planning to ensure that project milestones align with thesis timelines. As such, perhaps, it makes more sense for students to take on aspects of the project that are more guaranteed and adhere to specific deadlines. Co-production also requires overcoming institutional barriers such as rigid funding structures and mismatched priorities among partners (Laubenstein and Rummer, 2020). The process of co-developing research plans, collecting data, co-interpreting results and disseminating findings is inherently time intensive (a common theme noted by most co-authors). In conservation physiology, bridging the divide between physiological researchers and conservation managers can be particularly challenging, as evidenced by the need for ongoing engagement in projects such as the various case studies highlighted above. Without sustained commitment and flexibility, misaligned timelines—whether due to academic, regulatory or logistical constraints—can delay or even derail co-produced research efforts.

##### Multiple levels of approval are required

Co-production often requires ethical clearance and permits from multiple bodies. Difficulties can arise when committees have different viewpoints about various aspects of the project, and the additional need for approvals can delay the start of the research. Approvals may also be needed from various partner organizations including Indigenous governments. Developing formal agreements is important to manage expectations and ensure all parties are protected, but that takes time and can be complicated.

##### The social context in which projects occur can be complex

Partners working on the same project may have pre-existing interactions and relationships. This was particularly apparent in the salmon case studies. Salmon, and by extension the rivers they inhabit, have great cultural, ecological and economic significance, and have historically been a lightning rod for controversy and disagreements. Development of trust among the partners relies on acknowledgement and consideration of the social context in which the project occurs. Understanding how to navigate that space was enabled by ongoing social science studies (Young *et al.,* 2016; Reid *et al.,* 2022) to understand the diverse views of different participating groups.

##### The challenge of non-invasive sampling

Non-invasive sampling, which is common in conservation physiology, is not without its challenges and that can be magnified when doing co-production. For example, the small population sizes of the imperilled Wood Bison herds implicated in the Imminent Threat Assessment, has necessitated a hands-off approach and the application of non-invasive techniques for sample collection. The remote region and high fidelity of Wood Bison to seasonal habitats limit the window for sample collection, requiring capacity to be built-up and maintained between years. Due to the difficulties in sample collection historical samples are limited, and historical collection and preservation techniques may not be suitable for modern analyses. Therefore, it is important to develop questions based on feasibility and the availability of samples—and remain flexible when plans do not work out. That requires extensive communication with the partners involved in co-production which can itself turn this challenge into a benefit.

##### Results can change the path of the project

Co-production is a journey and when findings are interpreted collaboratively, it is possible that the project may change. As with the Nunatsiavut case study, which involved analyzing chemical contaminants in harvested species, a critical component among the team was addressing questions about animal health and food safety. An ongoing challenge in toxicology and the impacts it can have on human health is the lack of consumption guidance or eating guidelines for many of the oil-related contaminants in wild species. While part of this work did lead to a notice to harvesters released by the Nunatsiavut Government, having the human health risk assessment carried out in a timely fashion was a new priority in the project (as it often is until the contaminants data are in hand), and required additional data sharing and partners to be engaged. This meant that additional communications and processes needed to be added to the project, which required additional resources.

##### Clarity is required in terms of funding responsibilities

With potentially many partners involved in co-produced projects, there can be challenges with respect to determining funding responsibilities. As with the white rhinoceros case study, the research done was undertaken within South African National Parks’ Kruger National Park, using animals that belong to South African National Parks, their resources (such as helicopters and vehicles for animal capture, bomas for housing captive animals), and with support from the Park’s veterinary staff. It can be difficult in such a partnership to allocate research costs between partners. Early discussion and careful budgeting of total costs are required upfront with consideration being given to both in-kind and direct financial contributions. Done well, however, this activity can be hugely beneficial, as discussed above.

##### Sustaining long-term commitment and funding

Maintaining consistent funding streams and institutional support for a multi-decade research program requires ongoing demonstration of value to various stakeholders. The partnership must navigate different funding cycles from federal and state granting agencies and requirements of academic and conservation sectors while ensuring long-term research continuity. This challenge is complicated by the need to maintain both scientific productivity (to ensure further funding success) and practical conservation outcomes.

##### Balancing multiple stakeholder objectives

By definition, co-production involves multiple stakeholders (and possibly rightsholders) that may have numerous objectives. In the crocodile case study, researchers from The University of Queensland and crocodile experts from Australia Zoo share the common goal of crocodile conservation, each partner brings different priorities and requirements to the party. Critical to both partners is knowledge creation and an improved understanding of the ecology, physiology and behaviour of estuarine crocodiles. Publications are critical for the university researchers, especially for Ph.D. students and postdoctoral fellows, while for Australia Zoo dissemination of research findings to the general public and to key stakeholders (wildlife conservation decision-makers and politicians) is important. Similar balance was required between academic interests in the physiology and biogeography driving plant endemism in the PIlbara in Western Australia, and the dissemination of project-specific findings to key mining-industry stakeholders. Managing these diverse objectives while maintaining partnership cohesion requires mutual understanding and respect of each other’s priorities and objectives.

##### The challenges of working across scales

It is well documented that one of the inherent challenges with conservation physiology is working across biological scales, particularly moving from the individual organism to the population and then ecosystem (Ames *et al.,* 2020). There is also extensive physiological diversity within and among populations and species. These issues are not unique to co-produced research but rather are issues that need to be considered when co-producing projects. This is perhaps most salient when working with partners where the explicit goal is not simply to study an issue but rather to generate new knowledge to facilitate population or species recovery. In that context, it can be counterintuitive to focus on individuals (which is often the focus in conservation physiology research), while the functional management unit is the population or even ecosystem. The challenge lies is determining the extent to which it is important to understand the mechanisms underlying a problem (Cooke *et al.,* 2023) and working collaboratively to identify which endpoints and levels of biological organization make most sense.

#### Lessons

There are several lessons that emerged from our collective experiences and reflective case studies. We share those lessons here but acknowledge that there is no one ‘recipe’ for co-production. Indeed, co-production is inherently nuanced and bespoke to a given question, context and set of partners. Co-production itself is a journey and the co-authors on this paper have learned from both our successes and failures. These lessons are provided with the recognition that they are not intended to be prescriptive but rather informative.

##### Create space for diverse participants

Co-production works best when space is created for diverse participants. That was emphasized in the case study involving contaminants in Nunatsiavut where many partners were needed to explore questions from real-world exposure events to a suite of physiological metrics. The overall project was developed by Environment and Climate Change Canada staff with western trained toxicological knowledge, and staff from the Nunatsiavut Government’s environment department with environmental knowledge and training in both western and Inuit knowledge. Sampling in the communities was then carried out by Nunatsiavut Government conservation officers and community members that had local Inuit knowledge of the region. The development of the transcriptomics, epigenetics and metabolomics approaches that are compared to the analytical chemistry data was led by western trained academics and Environment and Climate Change Canada researchers, but always with the final approval of the work by Nunatsiavut Government. Ultimately, no one group from this project would have been able to carry out the depth and breadth of studies that have resulted from this collaboration. Knowledge co-production benefits from diverse experiences, including varied backgrounds, professions, socioeconomic statuses and cultures.

##### Focus on relationship building and the rest will (likely) follow

In its prioritization of novelty, Western science has often ignored the journey of research in favour of results. This disregards the importance of relationship building as a crucial component and neglected deliverable of research. Without prior established relationships, appropriate time needs to be allocated to projects to identify individuals, establish communications and gain trust. For example, the time it took to understand the diverse landscape of Wood Bison research and monitoring with partners was longer than expected. Relationships with collaborators have benefited the design and early research on Wood Bison conducted by the Environment and Climate Change Canada scientists. Benefits include contribution of expert perspectives, accessing samples and key contacts needed for the implementation and interpretation of research. This includes having knowledge of each other’s strengths, best highlighted by the white rhinoceros case study. The groups had interacted previously, through South African National Parks providing veterinary services for the academics’ research and had mutual respect and an understanding of the skills and expertise of each partner before engaging in discussion for co-production research projects. In the case of the crocodile case study, early successes established a strong working relationship, providing the foundation for, and confidence, in planning and undertaking a long-term research program. The success of co-produced conservation physiology projects depends not only on scientific expertise but also on the ability to engage meaningfully with stakeholders (Laubenstein and Rummer, 2020). Training in facilitation, mediation and cross-disciplinary communication is essential for ensuring that physiological research translates into actionable conservation outcomes. Developing these skills should be considered an integral part of training for conservation physiologists to foster more effective engagement with decision-makers, industry partners and Indigenous communities.

##### Embrace patience

Many projects that are co-produced take time to develop and evolve. In some cases, there are expectations of sampling over long time frames as was the case with the case study on *A. quadtrata,* which occurred over six years. The longer timespan served an important dual purpose: (i) it allowed for a quasi-adaptive approach in which results from early experiments informed the design of latter experiments and (ii) it provided time to build trust among the various collaborators, improving confidence in the research findings. A ‘slow science’ approach (Stengers, 2018) was valuable, because it allowed insightful experimental designs to emerge as one discipline (biogeography) informed the hypotheses of the next discipline (ecophysiology). Furthermore, the long timeframe allowed the partnership between management and research to fully digest the findings, exploring the implications and framing plausible responses in a way that fostered mutual respect and collaboration. Similarly, creating a long-term partnership allowed for ongoing research through multiple projects to progressively create an in-depth understanding of the adverse physiological responses in immobilized white rhino. It also promoted evaluation of intervention strategies not only within a research environment, but in clinical field settings where conditions can be quite different and variable. In turn, the outcomes of these field evaluations could be brought back to the research arena to inform the next generation of research questions.

##### Recognize that co-production can happen fast, when prior relationships exist

Although we often think of co-production being a slow process, in some circumstances, co-production can happen quite quickly. The Nunatsiavut case study stemmed from an emergency response situation where oil was spilled into the environment. While in many cases co-production is not a nimble process that happens rapidly, this case study highlights that when existing relationships are in place, co-production can be responsive and quick to implement. The spill happened around 9 June 2020, and within days the team had held several conference calls to discuss study objectives and research plans. This included some immediate sampling that was completed within a week of the spill and continued for several months and years following the spill. Notably, while these collections were fast, the ongoing dialogue about what analyses to run was iterative and ongoing.

##### Acknowledge that success is built upon teamwork

Teamwork and knowing the capabilities of team members were paramount in driving the success of the aforementioned crocodile research program, especially given that it is run from a very remote field location. Onsite there are two ‘teams’—a science team (the University of Queensland) responsible for attachment and implantation of the satellite and acoustic transmitters and a logistics team (Australia Zoo) responsible for the safe capture of estuarine crocodiles and running the Coolibah Research Station on the Steve Irwin Wildlife Reserve. A team leader for each crocodile captured was appointed and they made sure that everyone knew their role during capture. Safety was everyone’s responsibility. During any one field season, there are 4–5 team leaders that are rotated between crocodiles. Building in redundancy in terms of expertise is critical as personnel come and go over time, therefore training of team members to take on a leadership role happens most years.

##### Consider expanding and diversifying partnerships

Diversifying partnerships can provide new resources and opportunities. Expanding your network beyond the immediate species and location of interest can have immense benefits. For instance, applying a broader lens to Wood Bison health, instead of Wood Bison-specific questions, has allowed the team to address research gaps and collect samples that can provide information critical to the management of the at-risk herds. Collaborations with partners with access to farmed Wood Bison herds has also created opportunities to increase sample size and validate new methods and techniques. Even so, careful planning is critical to ensure that results are applicable and can be appropriately applied to the species and herds that are at-risk.

##### Develop a communication plan

Successful management of collaborative partnerships that include people from different academic and experiential backgrounds requires an effective communication strategy. The adopted approach should not only take cognisance of the different levels of expertise but also be able to effectively share often complex and specialized concepts, and strategies across these differences. It is important that a collective understanding is created of each partner’s roles and responsibilities to ensure a successful outcome to a research project. Although we do not have a good example to point towards, we encourage readers to check out Laubenstein and Rummer (2020) as a starting point.

##### Collaboratively identify biomarkers and endpoints that matter

Appropriate physiological endpoints are critical and can be dependent on co-production. While anaerobic recruitment and oxygen debt are suitable for assessing the immediate physiological impact of dam passage on fish, they do not directly address the question that is most relevant to the dam operators: What is the dam’s impact on population-level fitness of migrating adult salmon? To link events at the dam with survival to spawning grounds, telemetry experts collaborated closely with the First Nations organization that operates the spawning channel 50 km upstream from the dam.

## Conclusion

Conservation physiology has emerged as a mission-oriented discipline (Cooke *et al.,* 2013). As we are starting to document more ‘success stories’ in conservation physiology (Madliger *et al.,* 2016; Madliger *et al.,* 2021a,b), it is our collective perspective that those successes are in part attributable to a more fulsome embrace of the concept of co-production. Co-production has been widely touted for its role in generating actionable knowledge (Beier *et al.,* 2017) and identifying environmental solutions (Cooke *et al.,* 2020). When the lead author (Cooke) reached out to the conservation physiology community, he was not surprised by the extent to which co-production was being embraced as evidenced by the contribution of diverse case studies (spanning taxa, ecosystems, regions, types of partners and topics) that appear in this paper. There was clearly a critical mass of researchers doing co-production on projects involving vertebrates and especially salmonid fishes. Salmonids are widely known for their cultural and socio-economic value such that they are among the most studied animals on the planet but also among the most imperilled. Notably, salmonids are of great value to people which presumably necessitates and stimulates co-production. Only one of our case studies was on plants and none involved invertebrates, although this may represent biases and challenges in engaging conservation physiological research in these taxa (Madliger *et al.,* 2018). Despite quite different case studies, there was collective recognition that there were many benefits of engaging in co-production yet also a realization that there were also challenges with doing so. Importantly, we identified several lessons that can be considered by conservation physiologists when beginning to work on such efforts. As a diverse set of co-authors wearing many hats, co-production is a worthwhile endeavour and, as demonstrated in the case studies presented here, is helping to yield more success stories for species conservation.

There were a few elements of our guidance and experience that are particularly unique to conservation physiology mostly related to the conservation physiology toolbox. For example, given that physiological techniques often require lethal sampling, there can be conflicts when partners are uncomfortable with lethal sampling. Similarly, there may be concerns with non-lethal sampling methods or practical challenges with such work. In many ways, these are all issues that can be addressed by focusing on better/more communication as well as building trust and understanding. Indeed, some of the challenges that we identified here may in fact be overcome through effective co-production when diverse peoples and knowledge systems are embraced to tackle a given problem or challenge. Another important recognition is that co-production can lead to a variety of questions and thus requires building a team with the necessary disciplinary expertise to deliver answers. In that sense, co-production can be the stimulus for more interdisciplinary, cross-cutting and comprehensive research, which is something that has been touted as important in conservation science for many years (Kareiva and Marvier, 2012). We look forward to more case studies that demonstrate and share the successes and limitations of co-production that involve conservation physiology. We also acknowledge the need for the conservation physiology community to support each other on this journey and for more training and capacity building on how to engage in co-production in a respectful, ethical and meaningful way. As conservation physiology continues to evolve as a discipline, it is imperative that co-production becomes the norm rather than the exception (Laubenstein and Rummer, 2020). By institutionalizing co-production practices, conservation physiologists can bridge the knowledge–action gap and enhance the discipline’s impact on biodiversity conservation. We also acknowledge that co-production is just one element of the broader concept of ‘translational ecology’, which is explicitly focused on the intersection between knowledge generation and application (Schlesinger, 2010) and demands training specialists with capacity to do that work (Schwartz *et al.,* 2017). Having more conservation physiologists embrace career paths that focus on translational ecology (e.g. serving as knowledge brokers) would also help to bridge the gap and support those engaging in co-production.

## Data Availability

This is a perspective article and although this is based on various empirical studies, those data are published in the original source and here we discuss the process by which that research was conducted. As such, no data are provided here and readers are encouraged to consult the papers cited in each case study.
